# Exploring the Evidence Base for Acupuncture in the Treatment of Ménière's Syndrome—A Systematic Review

**DOI:** 10.1093/ecam/nep047

**Published:** 2011-06-23

**Authors:** Andrew F. Long, Mei Xing, Ken Morgan, Alison Brettle

**Affiliations:** ^1^School of Healthcare, University of Leeds, Leeds, LS2 9UT, UK; ^2^School of Community Health Sciences and Social Care, University of Salford, Salford, UK; ^3^Acupuncturist, Bolton, UK; ^4^School of Nursing, University of Salford, Salford, UK

## Abstract

Ménière's syndrome is a long-term, progressive disease that damages the balance and hearing parts of the inner ear. To address the paucity of information on which evidence-based treatment decisions should be made, a systematic review of acupuncture for Ménière's syndrome was undertaken. The method used was a systematic review of English and Chinese literature, from six databases for randomized, non-randomized and observational studies. All studies were critically appraised and a narrative approach to data synthesis was adopted. Twenty-seven studies were included in this review (9 in English and 18 in Chinese languages): three randomized controlled trials, three non-randomized controlled studies and four pre-test, post-test designs. All but one of the studies was conducted in China. The studies covered body acupuncture, ear acupuncture, scalp acupuncture, fluid acupuncture point injection and moxibustion. The studies were of varying quality. The weight of evidence, across all study types, is of beneficial effect from acupuncture, for those in an acute phase or those who have had Ménière's syndrome for a number of years. The review reinforces the importance of searching for studies from English and Chinese literature. The transferability of the findings from China to a Western context needs confirmation. Further research is also needed to clarify questions around the appropriate frequency and number of treatment/courses of acupuncture. The weight of evidence suggests a potential benefit of acupuncture for persons with Ménière's disease, including those in an acute phase and reinforces the importance of searching for published studies in the Chinese language.

## 1. Introduction

Ménière's syndrome is a long-term, progressive disease that damages the balance and hearing parts of the inner ear. It is most common between the ages of 40 and 50 and rare in children and onset after the age of 60. Its incidence is about 1 in 1000, equally distributed between men and women and well documented in Caucasian, African-American and Asian races [1]. The etiology and treatment of Ménière's disease is not fully understood. In a literature-based, clinical review of the diagnosis and treatment of Ménière's disease, Saeed [2] commented that “currently, the treatment of Ménière's disease is empirical. As yet, no treatment has prospectively modified the clinical course of the condition and thereby prevented the progressive hearing loss." Indeed, Thorp et al. [3] in a critical review of studies of medical and surgical approaches queried whether any evidence-based medicine existed in the treatment of Ménière's disease.

Conventional biomedical treatments include drugs, diet and surgery. Drugs often form the first line of treatment. These include: diuretics (to reduce the amount of sodium in the body); drugs to block symptoms of motion sickness, nausea and vomiting, and anxiety and vertigo; systemic or local corticosteroids to reduce inflammation within the inner ear and to stop any immune reactions; antibiotics; and drugs to improve blood flow in the inner ear [4]. The most commonly recommended dietary treatment is a low sodium diet. Patients have sometimes been helped by limiting certain components of their diet such as sugar, monosodium glutamate, caffeine and alcohol [4]. Surgical treatment is the last resort of conventional medicine for Ménière's disease, the most controversial [2] and of greatest risk.

As with other chronic diseases where treatment may have serious side-effects [5], or is variably experienced by patients as beneficial [6], or as with Ménière's disease there is limited high quality evidence as to the most appropriate treatments [3], some who suffer from the condition may explore the potential of complementary and alternative medicine (CAM) therapies to alleviate their symptoms. However, there is little published information on which CAM therapies might help those with Ménière's disease. To begin to address this evidence gap, and following anecdotal comments about the potential benefits of one CAM therapy—traditional Chinese medicine (TCM) acupuncture—a systematic review was undertaken of published evidence on the effectiveness of acupuncture in treating the symptoms of Ménière's disease, drawing on both English and Chinese language sources. This article reports the findings from that review.

## 2. Methods

### 2.1. Searching

As there was no previous review of published evidence in this area, and thus to ensure comprehensive coverage of the literature, any published English or Chinese language study, which used a quantitative outcome measure and had a sample size of 10 or more, was included. The criterion for sample size was set low in anticipation that current literature in this field might have a relatively small sample size; an *a priori* prescribed higher limit might unduly restrict the located evidence. The search was undertaken in two parts. The first was based on a search of English language literature, undertaken in 2004/-5 and forming the focus for a final year dissertation of an undergraduate student (K.M.) studying for a BSc in Traditional Chinese Medicine (Acupuncture) at the University of Salford; this was updated in 2009. The second comprised an opportunistic search of Chinese language literature as part of a study visit of one of the authors (M.X.) to China in the winter of 2003. The inclusion and exclusion criteria are summarized in Table 1.

Six databases were searched: MEDLINE via OVID interface (1966 to January 2009); The Cochrane Controlled Trials Register (on 18 January 2009), EMBASE (1974 to January 2009); CINAHL (1982 to January 2009); AMED (1985 to January 2009); and, for the Chinese language review, the Chinese database of Science and Technology (1993 to August 2003). A search of *eCAM* was also undertaken (on 6 January 2009). Hand searching of Chinese journals related to acupuncture and moxibustion was undertaken in China for the months of September to December 2003. Search terms of the electronic databases focused on terms related to *acupuncture* and *Ménière*'*s disease*. Free text search terms were used and controlled subject headings were exploded so that any subcategories were included (e.g., endolymphatic hydrops, acupuncture therapy or electroacupuncture). See the appendix for an illustrative search strategy.

### 2.2. Appraisal and Analysis

**Figure 1:**
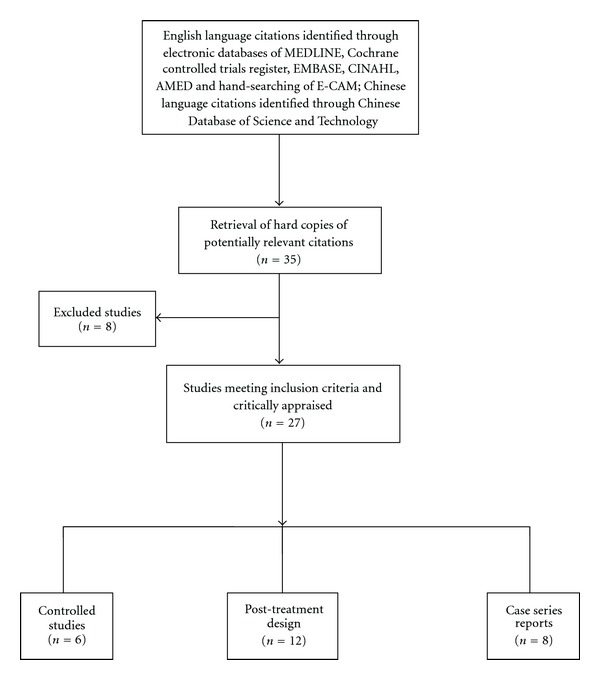
Flowchart literature search process.

Titles and abstracts were screened by one member (K.M. or M.X., respectively) and 27 papers were included in the review (Figure 1). All included studies were critically appraised. For the English language studies, an established evaluation tool [7] was used, suitably adapted to add in appropriate search-specific and acupuncture-related aspects [8]. For the Chinese language literature, data were extracted and simultaneously translated into English, using a standard template which included the following categories: study design; sample; treatment method; duration of treatment; outcome measurement; results; commentary on the appropriateness of acupuncture; and, overall comments/critique of the paper. Each English language paper was read and appraised by two members of the team independently (K.M. and M.X./A.F.L.) and any discrepancies resolved through discussion. The Chinese summaries were critically appraised (M.X. and A.F.L.) and where necessary further data extracted from the primary source.

A narrative approach to data synthesis was taken, with greater weight given to studies of greatest quality (minimizing bias, accuracy of diagnosis, appropriateness of acupuncture treatment and use of appropriate outcome assessment). A meta-analysis was inappropriate due to difference in study designs and outcome measures and the limited amount of quantitative data for individual patients. Restriction of the analysis to only controlled trials would have substantially narrowed the available evidence base and was judged inappropriate in light of the lack of previous attempts to draw together evidence on acupuncture for Ménière's disease.

## 3. Results

### 3.1. Nature of the Evidence

Of the 27 studies included in the review, 9 were studies in the English language and 18 in the Chinese language. All but one of the studies was conducted in China. The studies comprised: three randomized controlled trials (RCTs) [9–11]; three non-randomized controlled studies [12–14]; four pre-test, post-test designs [15–18]; nine post-test designs [19–27] and eight case series reports [28–35]. Together they comprised a total of 1888 patients in the acupuncture treatment arm. Seventeen of the studies followed the patients up for at least a year after the end of treatment.

Particular symptoms formed the focus of a number of studies, including vertigo [21] and dizziness [11, 18, 32]. The patients had been suffering from Ménière's disease for various periods of time, ranging from 1 to 24 years. Thirteen of the studies included patients at an acute phase, within 1–10 days of an acute attack. Three studies [9, 15, 30] explicitly focused on the effects of acupuncture for acute symptom relief. Sample sizes varied from 15 [11] to 286 [33], and 5 had a sample size of less than 30 [18, 19, 25, 26, 30]. The six controlled studies had total sample sizes ranging from 36 to 189 in the acupuncture arm. Nine of the studies had 50 or fewer participants.

The majority of studies employed a graded outcome measurement approach, differentiating three to four categories: “cured", “outstandingly effective", “effective/improved" or “not effective". “Cured" had the common meaning of “dizziness and other symptoms having disappeared" or “all symptoms disappeared", “able to return to work/resume normal activities" and “no recurrence within a 1 or 2 year period" (depending on the study's follow-up time). “Outstandingly effective" had a similar meaning but with recurrence of symptoms “occasionally", by, for example, 6 months. “Effective/improved" related to “relief of symptoms". Particular symptoms were often mentioned as part of the achieved outcomes, in particular dizziness and vertigo.

### 3.2. Nature of Acupuncture

The studies covered five types of acupuncture: body acupuncture; ear acupuncture; scalp acupuncture; fluid acupuncture point injection; and moxibustion. All such types are classifiable as falling within the TCM style, ensuring the “de-qi" sensation. Half of the studies involved an individualized TCM prescription approach to the treatment and half used a pre-set prescription, itself based on TCM principles and approaches to treating particular symptoms commonly experienced by persons with Ménière's disease, in particular, symptoms of dizziness, vertigo, nausea and vomiting. While none of studies mentioned the background of the acupuncturist, acupuncturists in China are all qualified medical doctors and at least 4 years training is required to work in a hospital.

No single, uniform number of courses or duration of treatment within each course was apparent. In two controlled studies [10, 14], once daily acupuncture was undertaken for 10 days; in another [13], no indication of the number of treatments or how often the treatment was given; in yet another [30], patients continued with the treatment until symptom relief. Across the studies, a treatment “once a day" for a course of “up to 10 sessions" with the possibility of a second (or more) course was common.

### 3.3. Quality of the Evidence

A number of weaknesses were evident in the studies. First, as only six of the studies had a comparison group and four others also included pre-treatment measurement, any observed improved outcomes could have arisen because of other factors, including symptom remission. Secondly, only eight of the studies either included audiometric testing [9, 19, 21] or recruited Ménière's disease patients according to established criteria for Ménière's [10, 18, 23, 24, 26]. Only two studies included audiometric testing as part of their outcome measurement [9, 27]. Thirdly, most of the Chinese language studies did not provide detail on the inclusion/exclusion criteria. Fourthly, very limited detail was provided on the choice of study participants or setting of the studies. Fifthly, the length of follow-up (and thus final outcome measurement point) varied; eight studies had a follow-up time of 2 years and nine had 1 year, while for seven no or little detail was provided.

### 3.4. Evidence of Effectiveness

The weight of evidence, across all study types, is one of the beneficial effects from five types of acupuncture—body, ear or scalp acupuncture, fluid acupuncture point injection, or moxibustion (Table 2). The three randomized controlled trials [9–11] demonstrate a statistically significant benefit of body or scalp acupuncture against Western medicine and vitamins, with a mean difference in the total effectiveness percentage of 14% in favor of acupuncture (*P* < .01). Two non-randomized controlled trials, comparing body acupuncture against Western medicine [12] or Chinese herbal medicine [13], show a similar mean difference in total effectiveness percentage (19%, *P* < .001). The evidence from the four pre-test, post-test studies reports even higher success rates (90–100%) for acupuncture, including moxibustion on acupoint Du-20, either on its own [18] or combined with body [15], and/or ear acupuncture [16] or herbal medicine [17]. High success rates are also reported from the post-test and case-series studies. As 13 studies included patients in an acute phase of Ménière's disease, the evidence suggests beneficial effects for both those in an acute phase and who have had Ménière's for a number of years. There is, however, insufficient evidence to recommend one, or another, type of acupuncture, nor the number of courses of treatment that might be needed for beneficial effect.

## 4. Discussion

This is the first systematic review of evidence on the potential benefits of acupuncture for the treatment of Ménière's disease and draws on both English and Chinese language sources. It demonstrates beneficial effects from up to five types of acupuncture (body, ear or scalp acupuncture, fluid acupuncture point injection, or moxibustion) and for both those in an acute phase and who have had Ménière's disease for a number of years. At the same time, it is important to note that the quality of evidence varies and is drawn from studies with varying potentiality for bias. In the five studies with a non-acupuncture comparison group (irrespective of the mode of allocation), the differences in total effectiveness percentage in favor of acupuncture were of the order of 14–19%.

Given that the weight of evidence is from China, further research within a Western context is required. There is also a need to clarify questions around the appropriate frequency and number of treatments or courses of acupuncture. This is especially important where persons have to pay for acupuncture treatment. Exploring possible sets of pre-defined acupuncture points is also important, given that not all acupuncturists in the UK or Europe adopt a TCM diagnosis and treatment approach [36].

The review differs in relation to others in the CAM area, as it draws on both English and Chinese literature. Such an approach led to the inclusion of 18 additional studies and, in principle (irrespective of study quality), strengthens the conclusions of the review. A similar conclusion is drawn by Yuan et al. [37] who explored treatment regimens of acupuncture for low back pain, accessing English and Chinese literature, the latter located by Chinese experts. Indeed, their systematic review identified substantive differences in the treatment frequency between practice in China and the West.

The inclusion of English and Chinese language papers does not however avoid the issue of publication bias; publications in other languages would still be excluded. Neither does it avoid publication bias arising from the greater likelihood of the reporting of positive results (i.e., the non-reporting of negative studies). As Moher et al. [38] argued, substantial bias may occur in the results of a CAM systematic review if languages other than English are excluded. Extending the searching process to the Chinese language is an important step, given the lengthy historical tradition and use of acupuncture within China. Indeed, accessing research literature published in the Chinese language perhaps needs to become a *sine qua non* in this type of literature review. This is challenging, requiring access to Chinese language readers [39, 40]. It was possible in this study only because one of the project team members (M.X.) was native Chinese. Optimal methods of accessing and including Chinese language literature however need further exploration.

Generalizing or transferring the results of studies undertaken in another country and cultural context is not straightforward. All but one of the studies took place in China. Citizens' attitudes to TCM and acupuncture in a Chinese cultural context, where there is a long tradition, recognition and establishment within the health care sector [36], along with established education and training approaches, are likely to be very different than in a Western European context. This has important consequences for patients deciding when to seek acupuncture. In China, acupuncture may be sought early or as a routine treatment. In the West, where the first line of treatment for someone with Ménière's disease would likely be biomedical, acupuncture may be sought only when the condition becomes chronic. There are also differences in education and training of, and access to, acupuncturists (traditional TCM trained practitioners or Western acupuncture [41], thus the recognized importance of reporting studies using the STRICTA framework [8]. This strongly suggests the need for a controlled trial of acupuncture, for both persons in an acute phase and those who have had Ménière's disease for a number of years.

Any such study needs to be appropriately designed. As there is no definitive, demonstrated bio-medical curative approach for Ménière's disease, choice of comparison group is not straightforward. A placebo design would seem both inappropriate and ethically problematic, as it is highly improbable that potential study participants will not be taking some medication or other approach to enable them to cope. While arguing for the need for greater and more rigorous evidence, Thorp et al. [3] make a similar comment in the context of conventional medicine. In Europe, an appropriate comparison might be drug treatment. In China, an appropriate contrast might be a (Western) drug approach or different forms of TCM approaches (e.g., types of acupuncture and/or herbal medicine). Indeed, the former form of comparison (acupuncture versus a drug or herbal approach) was used in four of the included controlled studies [9–12], and the latter (different forms of acupuncture or Chinese herbal medicine) in two others [13, 14]. It is important to note that choice of comparison group should be culturally determined.

Whatever choice is made, it is important that full methodological details are provided in the research report, utilizing the STRICTA recommendations [8] both as a guide to reporting but also study design. The review suggests the potential of exploring treatment “once a day" for a course of “up to 10 sessions" with the possibility of a second (or more) course. Such research should measure both the short- and longer-term symptom effect, based upon patient reports of symptom benefit and (time before any) recurrence and extent of severity/symptom relief at any recurrence, general health and well-being effects and a potential greater resolve to cope with the illness.

There are some limitations to this review. First, the Chinese literature search accessed only one database and limited hand searching. More studies might be generated from a wider search. Secondly, it is notable that the studies from China report positive effects of acupuncture. This may be due to publication bias, [42] or be a true effect, demonstrating that frequent and a large number of treatment sessions, evident in the Chinese studies, leads to better outcomes [37]. In this regard it is interesting to note one of the findings from a systematic review of clinical trials on acupuncture in the Japanese literature [43]; studies undertaken pre-1990 tended to focus on the appropriate choice of acupuncture technique, whereas post-1990 the focus tended to be on evaluation of acupuncture efficacy. This finding has some resonance within the Chinese literature in this review, especially within the case-series studies and their presentation and the depth of explanation about technique. Thirdly, inclusion of non-randomized and uncontrolled studies in the review could be argued to be problematic [44]. Such studies were used here as a means to support, or otherwise, findings from more powerful studies. In addition, such studies provided additional information on the maintenance of beneficial effects. Fourthly, in a recent report relating specifically to Cochrane systematic reviews of TCM, where only 5 of the 28 described RCTs could be authenticated as RCTs, Wu et al. [45] argue for the need for review authors to verify with study authors for claims that their RCTs were in fact randomized. This was not undertaken in this study. Fifthly, no attempt is made to draw together any of the TCM explanations indicated in the included papers about the mechanisms that might underpin acupuncture effecting the symptoms of Ménière's disease or other factors which may be associated with such symptoms.

## 5. Conclusion

The aim of this systematic review was to locate and critically appraise evidence for acupuncture as a treatment for Ménière's disease, drawing from both English and Chinese language literature. Despite the range in the quality of the located evidence, the overall conclusion is of the potential benefit of acupuncture for persons with Ménière's disease, including those in an acute phase. The review also demonstrates the importance of searching for studies in the Chinese language for such a therapy as acupuncture, given its lengthy historical tradition within Chinese medicine. As all but one of the studies took place within China, further research is needed in a Western health care context and to examine the frequency and number of treatment or courses of acupuncture.

## Figures and Tables

**Table 1:** Inclusion and exclusion criteria.

*Inclusion criteria*:
Study on patients with a diagnosis of Ménière's disease
All types of TCM acupuncture or moxibustion
Study using acupuncture or moxibustion in addition to other TCM, for example, herbal medicine
Studies of any controlled type and case series if the sample size was ≥10
Studies in the English or Chinese languages
*Exclusion criteria*:
Studies with a sample size of <10 or single case reports or reports of opinion
Studies which treated dizziness, tinnitus or other symptoms of Ménière's disease without a diagnosis of Ménière's
Other forms of Traditional Chinese Medicine without acupuncture or moxibustion

**Table 2:** Evidence table (English and Chinese language studies).

Study, study type and country	Treatment, sample size and follow-up time	Confirmed diagnosis and time with Ménière's disease	Appropriateness of treatment	Key findings	Summary evaluative comments and overall quality
Randomized controlled trials					

	Body acupuncture versus Western medicine 15-day treatment	MD patients attending hospital for acute attack	No TCM pattern diagnosis used; set acupoints; TCM rationale provided	Total effectiveness rate: 74% versus 49% (*P* < .05)	Strengths of the study include: confirmed MD, random allocation to treatment groups, detailed overview and rationale of the treatments, graded outcome measurements and audiometric testing. The short length of follow-up limits the generalization of the results.
Zhang et al. [9] China	(i) Acupuncture: once a day for 2-3 days; if symptom relief, then every other day;			69% versus 43% symptom control (*P* < .05)	
	(ii) Western medicine: two drugs (Serc and Vitamin B_3_) and Vitamin B_6_				
	(*n* = 76; *I* = 39; *C* = 37)	Unknown duration		Small, audiometrically confirmed, hearing change	
	
	Follow-up: 15 days (end of treatment period)				Overall quality judgment: Good

	Scalp acupuncture and Western medicine versus Western medicine and vitamin B_1_ and B_12_	MD confirmed by two medical committees	Set prescription without individualized diagnosis and treatment	Total effectiveness rate: 98% versus 89% (*P* < .05)	Strengths of the study include: confirmed MD, random allocation to treatment groups, detailed overview of the treatments, and graded outcome measurement. The chosen treatment approach is experimental, rather than a traditional TCM one.
Gao and Ni [10] China	(i) Acupuncture (or injected vitamins) once a day for 10 days as one course, total of three courses			89% versus 27% symptom control (*P* < .001)	
	(ii) Western medicine: once a day for 10 days, total of three courses	Up to 7 years			
	(*n* = 132; *I* = 58; *C* = 74)			84% versus 49% hearing improvement (*P* < .001)	
	Follow-up: 2 years				Overall quality judgment: Good

	Acupuncture and drug treatment versus drug treatment	Unclear if the MD cases confirmed MD	Individualized diagnosis and prescription	Total effectiveness rate for Ménière's group: 91% versus 50% (NS, *P* > .05)	Strengths of the study include: random allocation to treatment groups, individualized diagnosis, detailed overview of the treatments, and graded (and quantitative) outcome measurement. Weakness in very small number of MD sample size, non-confirmation of MD diagnosis and short follow-up time.
	(i) Acupuncture—key points; once daily (acute stage), once every 3 days (remission stage).				
Fang [11]* China	(ii) Drug treatment (sibelium, 10 mg once daily for 30 days)				
	(*n* = 65; *I* = 36; *C* = 29; of which Ménière's *I* = 11; *C* = 4)	1 day to 3 months (from dizziness)		7 out of 11 versus none of controls dizziness cured	
	Follow-up: 30 days				Overall quality judgment: Fair

Non-randomized controlled trials					

	Acupuncture versus Western medicine and vitamin	Unclear if confirmed MD	Treatment group has TCM diagnosis and individualized treatment; control group has set acupoints.	Total effectiveness rate: 100% versus 74% (*P* < .01)	Strengths include details over outcome measurement; length of follow-up; use of TCM pattern differentiation. Weaknesses include: lack of clarity over how patients are allocated to the two treatment groups; MD not confirmed; and limited detail over treatments (form or frequency).
	(i) Acupuncture—key and specific points.			60% versus 39% cured and no recurrence at 2 years (*P* < .01)	
Yu and Shi [12] China	(ii) Western medicine (vitamin C and Luminal (an anti-convulsive drug) injection)	1–7 years		30% versus 8% outstanding improvement and no recurrence at 1 year	
	No details given on daily frequency or number of sessions of acupuncture (*n* = 168; *I* = 86; *C* = 82)				Overall quality judgment: Fair
	Follow-up: 2 years				
	Body acupuncture versus Chinese herbal medicine (for vertigo)	Patients with MD	No TCM pattern diagnosis used to choose acupoints	Total effectiveness rate: 99% versus 88% (*P* < .01)	Short report, but adequate details on MD confirmation, treatment points, appropriate 2:1 case: comparison subject ratio and explanation of set acupoint prescription. Lack of detail over how patients were allocated to the treatment and comparison group (non-random).
	(i) Acupuncture: Two courses of 15 sessions 1 p day, with 5 day rest			93% versus 60% “complete" response (*P* < .001)	
Yan [13]* China	(ii) Chinese herbal medicine: Two courses of 15 sessions 1 p day, with 5 day rest	102 patients had MD for ≤1 year, 135 1–5 years and 20 > 5 years	Set acupoint prescription	99% versus 88% “complete" or “partial" response	
	*n* = 257; Ac = 189; Hb = 68			11% versus 49% recurrence rate (*P* < .001) (when not indicated)	
	Follow-up: 1 year				

	Scalp and ear acupuncture versus body acupuncture	Confirmed MD by hospital	Set prescription without individualized diagnosis and treatment	Total effectiveness rate: 94% versus 73% (*P* < .01)	Strengths include details over acupuncture treatment and their rationales and outcome measurement.
Qin and Jia [14] China	(i) Scalp and ear acupuncture once per day for 10 days, plus patients (taught to) self-treat for 5 days.			53% versus 33% cured (*P* < .01)	Weaknesses include: lack of follow-up; and lack of clarity over how patients are allocated to the treatment groups.
	(ii) Body acupuncture once a day for 10 days	Duration—no information		27% versus 23% marked improvement	
	(*n* = 102; *I* = 72; *C* = 30)				
	Follow-up: none				Overall quality judgment: Fair

Pre-test, post-test design					

	Acupuncture	Patients with MD in acute phase (all previously treated with Western medicine without effect)	Set prescription, needling one point (Gang Shen), based on clear rationale	Total effectiveness rate: 100%	Extensive details over treatment procedure and rationale are provided (Gang Shen is an experimental point for MD). But lack of detail over choice of participants and outcome measurement scale.
Li and Li [15] China	Once a day for 20 days			77% acute symptom control after one treatment; 16% after two.	
	*n* = 56			Most patients needed only 7 sessions to experience benefit.	Overall quality judgment: Fair
	Follow-up: 1 year	Up to 8 years			

Li [16] China	Herbal medicine and ear acupuncture, for 2-60 days	Patients with MD according to explicit criteria	Individualised treatment following TCM principles for herbal medicine; ear acupuncture points set in conjunction with herbal medicine	Total effectiveness rate: 90%	Strengths include: clear diagnostic criteria, appropriate TCM principles and outcome measurement in relation to these principles and descriptions. But lack of detail over treatment duration or choice of participants.
	*n* = 90	Up to 20 years		72% cured	Note: this is predominantly a herbal approach with ear acupuncture as an adjunct.
	Follow-up: 1 year			18% improved	Overall quality judgment: Good
	Scalp acupuncture plus herbal medicine Once daily acupuncture for 7 days; simple herbal tea (to continue tea for 1 year)	MD acute stage (hospitalized following acute attack)	TCM individualized diagnosis and treatment, with herbal remedy adjusted	Total effectiveness rate: 97% 18% outstanding effect	Strengths include: clarity over outcome measurement, TCM diagnosis and treatment and length of follow-up.
Dong and Zhou [17] China	*n* = 180			70% cured	
	Follow-up: 1 year	Up to 10 years		8% improved	Overall quality judgment: Good

	Moxibustion at Du-20	Patients with clinically confirmed MD	TCM individualized diagnosis with set acupoint	Total effectiveness rate: 100%	Use of standard diagnosis criteria and outcome measurement criteria for MD, appropriate acupoint with TCM rationale. (Note: treatment also included advice on diet and emotion) Overall quality judgment: Good
Sun and Li [18] China	Twice a day for 15 days			75% cured	
	*n* = 20	Up to 2 years		25% outstandingly improved	
	Follow-up: 1 year		(Du-20)		

Post-test design					

	Electro-acupuncture, acupuncture and moxibustion	MD patients who had auditory vertigo symptoms	One set point for acupuncture, another for moxibustion; other points added according to TCM diagnosis	Total effectiveness rate: 100%	Targets only cases with auditory vertigo syndrome. Detailed description of other Ménière's symptoms of cases; full explanation of potential role of acupuncture in treating Ménière's.
Dai and Liang [19]* China	One treatment daily for up 6 days (30 min application)			70% “cured"	
			
	*n* = 23			18% “excellent"	
	Follow-up: 1 year	Up to 17 years			
				

	Acupuncture	MD—query over criteria	Individualized treatment following TCM principles	Total effectiveness rate: 90%	Strengths include: appropriate TCM principles. But lack of detail over treatment duration, choice of participants and diagnostic criteria for MD.
	10 sessions for 1 month			22% cured	
Liu [20] China	*n* = 51	Duration—no information		69% improved	
	Follow-up: 2 year				Overall quality judgment: Poor

	Moxibustion at Du-20 (over 2 hours), for up to three sessions 32	Confirmed MD (Otol. Dept)	Du-20 is a good experiential point for dizziness.	Total effectiveness rate: 100% 100% symptom relief (8 from one session, 10 from two, and 14 after three).	Strengths include: MD confirmed by Otolaryngology Department, use of standard treatment duration, use of an appropriate (Du-20) treatment point and length of follow-up.
Chao [21] China	*n* = 32		Not diagnosed according to TCM theory.		Weaknesses include: unclear sampling criteria, sole focus on one MD symptom (dizziness) and lack of control group.
	Follow-up: 2 years	average 9 years	Note: moxibustion only	No recurrence after 2 years.	Overall quality judgment: Fair
	Electro-acupuncture	Patients with MD	Treatment according to TCM pattern diagnosis	Total effectiveness rate: 74%	Short article; in consequence, limited details on methods, except on acupuncture treatment.
Tian [22]* China	Two to three courses of 10 sessions daily, with 2 days rest between course (10–20 min application)	Fifty had MD for 1–5 years, 22 for >5 years		50% “marked improvement"	
	*n* = 72			24% “improved"	Overall quality judgment: Fair
	Follow-up period: 1 year			Treatment more effective in cases of shorter duration	

	Acupoint injection at Du-20	Patients with MD according to “book" of common diseases	Set, single point used, with TCM rationale	Total effectiveness rate: 100%	Clear diagnostic criteria with detailed treatment procedure. But duration of follow-up is not stated and lack of clarity over outcome measurement (causes of dizziness disappeared).
Bo [23] China	Once a day for 10 days, 1 day break, then another course of treatment (3–30 sessions in total)			75% cured	
	*n* = 88				Overall quality judgment: Fair
	Follow-up: not stated	Up to 3 years		23% improved	

Wang and Chen [24] China	Acupuncture points injection with Dansheng liquid on one side at one time plus scalp acupuncture	Patients with MD	Set prescription but based on good TCM treatment principles	Total effectiveness rate: 92%	Combined traditional and scalp needling plus points injection seems a potentially good treatment method, and based on TCM principles. While diagnosis is based on TCM principles, treatment is not individualized. Good detail on treatment procedures.
	Acupuncture with injection once a day, with scalp acupuncture every other day, for 10 days. 5-day break, second course of treatment	Up to 14 years		62% cured	
	*n* = 50			30% outstandingly improved	Overall quality judgment: Fair
	Follow-up: 2 years				

	Body acupuncture	Patients with MD	Treatment according to TCM pattern diagnosis	Total effectiveness rate: 94%	Study report is from an abstract, translated from the original Chinese paper. Brief detail on methods is provided, with extensive detail on needling and TCM rationale.
Zhang [25]* China	Courses of 10 sessions with a 2-day rest between (30 min application)			72% cured	
	*n* = 18	Up to 6 years.			
	Follow-up period: 2 years			22% marked effect	Overall quality judgment: Fair
	Acupuncture	MD—query over criteria	TCM pattern differentiation	Total effectiveness rate: 100%	Lack of detail over choice of participants or diagnostic criteria for MD. While diagnosis is individualized, the treatment points are only for one TCM diagnosis pattern. It is unclear if the treatment is individualized.
Zhou [26] China	3–15 sessions, once per day (average of 9)				
	*n* = 20	Up to 8 years		80% cured	Overall quality judgment: Poor
	Follow-up: 1 year			20% improved	

	Acupuncture	MD—query over criteria	Set acupoints based on explicit TCM rationale for point choice	Total effectiveness rate: 92%	Strengths include: explicit rationale for set acupoint prescription, based on TCM theory, and extensive details over treatment methods. Lack of clarity over how many courses were provided and diagnostic criteria.
Zhang [27] China	Once a day for 10 days, 5 day break, another course			60% cured	
	*n* = 60	Up to 10 years		30% outstandingly improved	Overall quality judgment: Poor
	Follow-up: 2 years				

Case series					

	Body acupuncture, ear acupuncture and moxibustion (and, rarely, scalp acupuncture)	Patients with MD—with confirmed auditory tests	Treatment according to TCM pattern diagnosis	Total effectiveness rate: 100%	Very brief report on cases over 5-year period, with limited detail on outcome measurement (focus on vertigo). Strengths include: the use of audiometric tests (for auditory acuity) and extensive detail on needling and TCM treatment rationale.
Steinberger and Pansini [28]* ?Yugoslavia	Five treatments, once a day (30 min application)				
	*n* = 34			100% success for vertigo after 3 treatments	Overall quality judgement: Poor
	Follow-up period: unclear				

	Body acupuncture	Patients with MD according to clinical symptoms	Set acupoint prescription, with extra points if tinnitus and deafness were severe	Total effectiveness rate: 93%	Reporting on 18-year case series of clinically confirmed MD cases. Short report, with limited but adequate detail on the acupuncture treatment.
Xu and Ge [29]* China	Courses of 10 sessions, once a day (20–30 min application)			39% cured	
				33% “marked" improvement	
	*n* = 75	18 had had MD for <1 year, 57 for ≥1 year		21% improved	
	Follow-up period: 1 year			On average, needed two treatment courses (range 1–5)	Overall quality judgment: Poor

	Body acupuncture	Patients with MD, with a “sudden onset" of symptoms	Treatment according to TCM pattern diagnosis	Total effectiveness rate: 100%	Brief report on cases treated since 1975 with very limited detail, except on rationale and approach to treatment (and ways to improve the treatment).
Tian [30]* China	10–15 treatments			100% “cured" (Eight had alleviation of symptoms after four treatments; 13 after 7)	
	*n* = 21				
	Follow-up period: unstated				Overall quality judgment: Poor
	Acupuncture	MD—query over criteria	Treatment following TCM principles, but only for the one symptom of dizziness	Total effectiveness rate: 99%	Strengths include: treatment based on appropriate TCM principles. But lack of detail over choice of participants or diagnostic criteria for MD, treatment not individualized, no standard course of treatment, and unclear length of follow-up
Song and Yi [31] China	Once a day until all symptoms disappear (mean *n* = 5; range 2 to >10)	Up to 15 years		91% cured over short term	
	*n* = 152			8% improved	Overall quality judgment: Poor
	Follow-up: unclear				

	Acupuncture	Patients with MD at an acute stage	No TCM diagnosis differentiation or individualized treatment	Total effectiveness rate: 98%	Following appropriate TCM principles for treating two symptoms (dizziness and vomiting) of MD, but limited detail and no explicit follow-up mentioned.
Zhu [32] China	*n* = 51	Up to 6 years		64% cured	
	Follow-up: not indicated			32% outstanding effect	Overall quality judgment: Poor

	Acupuncture	Patients with MD	Appropriate acupuncture at single point. No TCM pattern differentiation	Total effectiveness rate: 90%	The study reports on a large group of patients; the treatment provided is an integrated treatment for MD, but there is a lack of detail over the number of courses given.
Zhang and Shang [33] China	Once a day for 3 days (as one course)			57% cured	
	*n* = 286	Up to 24 years		28% outstanding effect	
	Follow-up: 1 year				Overall quality judgment: Fair

	Body acupuncture and moxibustion	Patients with MD	Treatment according to TCM pattern diagnosis	Total effectiveness rate: 100%	Short report on cases treated over a number of years. Argues that Ménière's syndrome belongs to the category of “dizziness" in TCM. Strengths include one year follow-up and clear rationale for treatment.
Lu [34]* China	One treatment per day (30 min application); 2–14 daily treatments given (mean of 7.4)	Twenty-two patients had MD ≤10 years; eight for >10		87% cured	
	*n* = 30			13% effective	
	Follow-up: 1 year			30% symptom free after 1 year; 47% re-occurrence within 6 months	Overall quality judgment: Fair

	Acupuncture plus moxibustion at Du-20	MD—query over criteria	TCM pattern differentiation and treatment	Total effectiveness rate: 100%	Reporting on cases treated since 1988 for TCM diagnosis of dizziness.
Wang [35] China	Once a day for 7 days as one course of treatment				
	*n* = 30	Up to 10 years		97% cured	Overall quality judgment: Fair
	Follow-up: 2 years				

Asterisk indicated studies located from English language search. RCT: randomized controlled trial; CT: controlled trial; MD: Ménière's Disease; I: Intervention; C: Comparison/Control.

## Appendix

### Search Strategy for MEDLINE via OVID interface

1. meniere$.mp.
2. (endolymphatic adj hydrops).mp.
3. exp Endolymphatic Hydrops/
4. exp Meniere Disease/
5. 1 or 2 or 3 or 4
6. exp Acupuncture Therapy/
7. (acupuncture or electro? acupuncture or auriculotherapy or (auricular adj needles) or (needling adj therapy) or (electric adj needling)).mp.
8. exp Medicine, Chinese Traditional/
9. 6 or 7 or 8
10. 9 and 5

Key:

mp = title, original title, abstract, name of substance word, subject heading word
exp = explode subject heading
/ = subject heading (MESH) search
$ = truncation symbol
? = wildcard symbol
adj = adjacent to
